# A Novel Polymeric Membrane Sensor for Chlorhexidine Determination

**DOI:** 10.3390/s23239508

**Published:** 2023-11-29

**Authors:** Joanna Lenik, Karolina Sokal

**Affiliations:** Department of Analytical Chemistry, Institute of Chemical Sciences, Faculty of Chemistry, Maria Curie-Sklodowska University, Maria Curie-Sklodowska Square 3, 20-031 Lublin, Poland

**Keywords:** potentiometry, solid contact, ion-selective electrode, heptakis (2,3,6-tri-O-benzoyl)-*β*-cyclodextrin, chlorhexidine determination

## Abstract

In the present work, potentiometric sensors with polymer membranes used for chlorhexidine (CHXD) determination were developed. The polymer membranes were plasticized with bis(2-ethylheksyl)sebacate (DOS) or 2-nitrophenyloctyl ether (*o*-NPOE). The active compounds used in the membrane were cyclodextrins, crown ethers, and ion exchangers. The best-constructed electrode was based on neutral heptakis(2,3,6-tri-O-benzoyl)-*β*-cyclodextrin with lipophilic salt (KTpClBP)—potassium tetrakis(4-chlorophenyl) borate—dissolved in plasticizer, DOS. The presented electrode is characterized by an average cationic slope of 30.9 ± 2.9 mV decade^−1^ within a linear range of 1 × 10^−6^ to 1 × 10^−3^ mol × L^−1^, while the value of the correlation coefficient is 0.9970 ± 0.0026. The response time was about 5 s when increasing the sample concentration and about 10 s when diluting the sample. The electrode potential is independent of the pH within a range of 4.0–9.5. The polymeric membrane sensor was successfully applied for assays of chlorhexidine digluconate in pure samples and pharmaceutical samples. The relative error from three replicate measurements was determined to be 1.1%. and the accuracy was RSD = 0.3–1.1%.

## 1. Introduction

Ion-selective electrodes are membrane sensors whose development continues to progress in the determination of medicinal substances both in drug samples and in body fluids, such as blood serum and urine. Clinical analyses of drugs and the determination of their concentration in biological specimens are very important and medically significant. Such tests are performed before a drug is introduced to the market; i.e., it must undergo appropriate phases of preclinical tests in vitro and then in vivo. At this stage of research, measurements of drug concentrations in various tissues and body fluids of animals provide information about the pharmacokinetic profile of the tested molecule in the animal body, which allows for a preliminary determination of the drug’s behavior in the human body and a very general view of its effectiveness and safety profile. 

On the other hand, research in the field of drug quality control is related to chemical assessments of the quality of medicinal substances in terms of their suitability for preparing drugs and the needs of pharmacotherapy. A substance with medicinal properties must meet strictly defined standards to guarantee its quality. Electrochemical sensors are such simple devices that they can be freely used for the quantitative analysis of pharmaceutical compounds in complex samples, i.e., pharmaceutical preparations. Such sensors offer many advantages: simplicity of construction, reversibility, reproducibility, repeatability of measurements, wide linear range, low limit detection, high selectivity, precise, accurate analytical information, and the relatively low cost of sensor manufacture and overall analysis. Potentiometric membrane electrodes have many conveniences over other analytical instruments, which contribute to the intensive development of research. This is evidenced by numerous studies in the literature, including review articles [1,2].

Recently, potentiometric chemical sensors have been prepared and investigated for the determination of compounds such as isoniazid, gabapentin, lidocaine, cysteamine, pyridoxine, memantine, and fluconazole [2]; alogliptin, saxagliptin, and vildagliptin [3]; tetracycline HCl and metronidazole [4]; safinamide [5]; mephedrone [6]; and many others. The developed ISEs were successfully used for analytical determinations of pharmaceutical and natural samples with high recovery factors.

One of the medicinal substances belonging to the antiseptic group is chlorhexidine. Given the COVID pandemic that has spread around the world, this group of sanitizers and disinfectants has become very widespread. Chlorhexidine is a guanidine derivative and has bacteriostatic and bactericidal properties. It dissolves less easily in water and more easily in alcohols. It is most often used in the form of salts that are easily soluble in water. Chlorhexidine salts, digluconate, acetate, and hydrochloride do not irritate mucous membranes and skin, and therefore, they are used in preparations for disinfecting hands, washing the surgical field, and disinfecting room tools. Additionally, they are used in wound disinfectants, mouthwash, toothpaste, tablets, and nose and eye drops [7]. Chlorhexidine is widespread, especially in dentistry. Antimicrobial properties (for instance, CHXD reduces the concentration of the bacterial strain *S. mutans* in saliva and plaque and destroys the bacteria *E. faecalis* located in dentinal tubules) allow this compound to be used in mouthwashes at a concentration of 0.2% and in the form of 1–2% gels in the treatment of root canals and cavities. 

**Figure 1:**
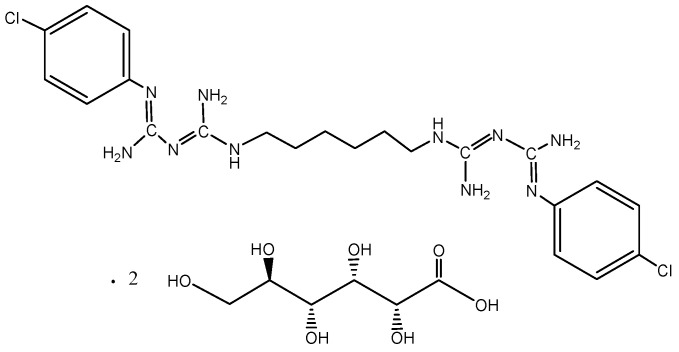
Bis(p-chlorophenyl) diguanidohexane digluconate.

The systematic name of chlorhexidine is 1,1’-hexamethylene-bis-[5-(p-chlorophenyl)biguanide]. The chlorhexidine molecule is axially composed of two 4-chlorophenols rings and two guanidine groups, which are connected by a hexamethylene chain (Figure 1). Chlorhexidine digluconate is the compound most commonly used in disinfectant fluids. It is impossible to obtain a solid form of chlorhexidine digluconate, so this salt is produced as an aqueous, colorless solution in a concentration of 20% because, in higher concentrations, it is too viscous and uncomfortable to use. Its solubility in water is good, and it is also soluble in ethanol at a ratio of 1:3 (in 70% alcohol) or 1:5 (in 96% alcohol) [8].

Summing up, through its activity against both Gram-positive and Gram-negative microorganisms and relatively low toxicity, chlorhexidine has a wide range of applications in human and veterinary medicine and dentistry. A large variety of products makes quality control, pharmacodynamic analytical tests, and pharmacokinetic studies necessary and still valid. This is evidenced by numerous scientific works, such as a review article from 2010 [9] that describes methods to analyze, in great detail, CHXD, involving spectrometry, chromatography, and colorimetric reaction. In modern analytical laboratories, there is a need for significant stability in analysis techniques, and therefore, analytical methods are continuously changing and developing. Over the past dozen years, numerous papers describing these methods have appeared, such as reversed-phase high-performance liquid chromatography [10,11,12], liquid chromatography coupled to tandem mass spectrometry (LC–MS/MS) [13,14,15], spectrophotometric methods [16,17], and electrochemical methods [18,19,20]. The Polish Pharmacopoeia [21] recommends titration in anhydrous acetic acid medium with perchloric acid at 0.1 mol L^−1^. The titration completion point is determined with the potentiometric method. Most of the above methods involve long and rather complicated sample preparations for analysis (converting analytes into suitable derivatives with properties that enable their determination or extraction steps) and require the use of high-cost apparatuses. Therefore, there is a need for the development of an inexpensive selective tool for the determination of the analyte. Analytical methods based on electrochemical sensors can be treated as a good alternative to the abovementioned methods. Such tools are simple, cost-effective devices. The design and development trends in such sensors, both voltammetric and potentiometric ones, are diverse. Nowadays, modified electrochemical sensors have been introduced, especially solid contact electrochemical sensors (SCESs). The conductive material can be gold, platinum, or carbon graphite in the form of glassy carbon, pyrolytic graphite, or semiconductors, i.e., indium oxide and tin. A variety of materials are used to modify the surface of electrodes, including carbon nanotubes, metal oxides, conductive polymers, and inorganic catalysts. There are many simple modification methods used in the construction of electrochemical sensors. The layer-by-layer (LBL) self-assembly technique provides a simple and unsophisticated method for the construction of stable nanostructured thin films. Self-assembled monolayer (SAM) techniques provide an easy way to modify the surface of metal electrodes with various active compounds modified with organosulfur compounds (thiols, disulfides, silanes). In addition, modified carbon paste electrodes, screen-printed carbon electrodes (SPCEs), and solid-contact PVC-based polymeric membrane electrodes are very common [22,23,24,25]. Two ion-selective electrodes have been reported to be used in the quantification of CHXD. Carbon screen-printed electrodes (with and without graphene) with a polymer membrane containing potassium tetrakis (4-chlorophenyl) borate, 2-hydroxypropyl-β-cyclodextrin [26], and other graphite disc electrodes based on the CHX-TBP ion pair have been developed [27]. 

This paper describes Ag/AgCl solid contact electrodes for chlorhexidine determination. The construction of the sensor was simple and low-cost and was published in earlier papers and in the chapter of a book [28,29,30]. The novelty of this work consisted of the incorporation of other active substances belonging to macrocyclic compounds, which improved some parameters of the developed electrodes. Furthermore, no analytical literature detailing a sensor for chlorhexidine determination with identical design and analytical parameters has been found. The proposed method enables a selective, sensitive, and precise determination of chlorhexidine in pharmaceutical samples that is also cost-effective and convenient. 

## 2. Materials and Methods

### 2.1. Chemical and Reagents 

All chemicals and reagents were of analytical grade. The components of the membrane included bis(2-ethylheksyl)sebacate (DOS) from Merck Schuchard (Hohenbrunn, Germany); 2-nitrophenyloctyl ether (*o*-NPOE) from Fluka (St. Gallen, Switzerland) emulsion PVC from Tarwinyl (Tarnów, Poland); and heptakis (2,3,6-tri-O-benzoyl)-*β*-cyclodextrin (HSB*β*CD), dibenzo-18-crown-6 (DBC-6), sodium tetrakis [3,5-bis(trifluoromethyl)phenyl]borate (NaFBP), and potassium tetrakis(4-chlorophenyl)borate from Sigma-Aldrich (St. Louis, MO, USA). The chloride salts of interferents were obtained from Fluka (St. Gallen, Switzerland). Additives (excipients) like trisodium citrate and sodium dodecyl sulfate (SDS) were purchased from Fluka (St. Gallen, Switzerland); D-mannitol, glucose, glycerol anhydrous, propylene glycol, and sucralose were obtained from Chempur (Piekary Śląskie, Poland); and xylitol was from Sigma. Chlorhexidine digluconate solution (20% *w*/*v*) was from Alfa Aesar (ThermoFisher GmbH, Braunschweig, Germany). The other reagents used in the work included tetrahydrofuran (THF) (Merck); acetic acid, sodium acetate, and sodium hydroxide from POCh (Gliwice, Poland) and pharmaceuticals; Corsodyl (chlorhexidine digluconate) 0.2% *w*/*v* liquid mouthwash (GlaxoSmith Kline Consumer Healthcare, Brentford, UK); and Eludril Extra (chlorhexidine digluconate) 0.2% *w/v* liquid mouthwash (Pierre Fabre, Castres, France).

A stock solution of chlorhexidine digluconate of concentration 10^−2^ mol L^−1^ was prepared via the dilution of 20% CHXD solution into a 100 mL volumetric flask and subsequently to the mark with deionized water. Working calibration solutions of concentration 10^−7^–10^−3^ mol L^−1^ were prepared from it via rigorous dilution with the same water. All aqueous solutions were prepared with deionized water of conductivity 0.07 μs/cm (Elix Advantage System Milli- Q plus Millipore, Spittal an der Drau, Austria).

### 2.2. Apparatus and Measurement 

Electromotive force measurements of the ion-selective chlorhexidine electrode and the reference electrode were performed at 22 ± 1 °C. The Orion 90–02 Ag/AgCl reference electrode salt bridge was filled with 1 mol L^−1^ CH_3_COOLi. The measurements were made using the Electrochemistry EMF Interface system (Lawson Labs. Inc., Malvern, PA, USA) and an IBM PC computer. The electrodes were conditioned for 24 h in 10^−3^ mol L^−1^ CHXD solution before the first measurement to saturate the membrane with the main ion of chlorhexidine. Because the electrodes were kept in dry air, before each subsequent measurement, they were conditioned (soaked) in the main ion solution (10^−4^ mol L^−1^ CHXD) for 15 min to bring them to a state where ion exchange on the membrane surface would be possible. The experiments involved stirring the solutions using a magnetic stirrer and recording the potential (±0.5 mV) after stabilizing. 

The pH was determined using a Thermo Orion (NTL, Warsaw, Poland) 81–72 glass electrode and an Elmetron CX721 (±0.1 mV) (Zabrze, Poland) multifunction computer from Poland.

### 2.3. Electrode Construction and Preparation of the Membranes

**Figure 2:**
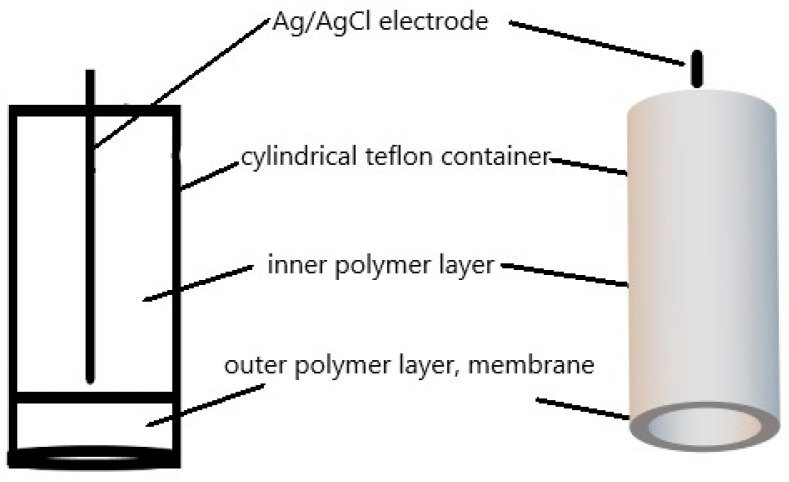
Scheme of polymeric membrane sensor.

The electrode’s main component is a polymeric membrane phase that directly contacts a silver–silver chloride electrode (Figure 2). It rests inside a cylindrical Teflon container that is screwed onto the electrode frame, while the body of the electrode is made from insulating material. The membrane phase includes two layers: an inner one and an outer one. An Ag/AgCl reference electrode is placed in the inner layer, creating a homogeneous system comprising the plasticizer and PVC. The reference electrode’s potential in the system is constant, as determined by using chloride ions from PVC degradation and AgCl dissociation and dissolution in the plasticizer [31].

The outer layer, which is in contact with the solution to be analyzed, contains the active ingredient in addition to the components of the inner layer. The inner layer of the membrane is produced via gelation, where PVC is dissolved in the plasticizer via ultrasound. The mixture is used to fill the Teflon sensor up to the coating of the reference Ag/AgCl electrode after being vacuum-deaerated at room temperature. The complete solution is then placed in the Teflon holder of the electrode sensor. The gelation should then take place at an elevated temperature of about 100–120 °C for about 30 min, depending on the specific type of plasticizer used. The outer layer is formed via evaporating tetrahydrofuran. Next, the mixture is applied in drops directly to a pre-gelled internal coating and left to evaporate. This is where plasticization occurs below the glass transition temperature of the PVC. The constructed electrodes with solid contact exhibit reproducible and stable potential over time. The electrodes are stored in air during the measurements.

### 2.4. Determination of Chlorhexidine in Pharmaceuticals

Two commercial pharmaceutical formulations, Corsodyl and Eludril Extra, 0.2% *w/v* (liquid mouthwash), containing chlorhexidine digluconate were analyzed using the chlorhexidine selective electrode. The liquid samples were prepared by measuring predetermined volumes and diluting them in the acetate buffer solution with a pH of 4.5.

The solutions of the samples (2.2 × 10^−4^ mol L^−1^) were prepared by transferring 5 mL of the Corsodyl and Eludril solution into two separate volumetric flasks and filling to the mark with acetate buffer solution. The respective solution (20 mL) was transferred to a 50 mL beaker, and the chlorhexidine electrode was immersed with the reference electrode. The potential of the electrode was measured. It was compared with the calibration curve. As an alternative, the potentials were measured before and after the addition of 2 mL of chlorhexidine standard solutions (c = 2.2 × 10^−3^ mol L^−1^) to the sample solution or after the addition of four times the standard. The calibration curve, the standard addition method, and Gran’s method were used to calculate the unknown concentration of chlorhexidine [32].

## 3. Results

### 3.1. Composition of Membrane and Electrode Performance

The ionophore, called the active substance, is known to be the most important part of the ion-selective membrane. Most of the properties of the electrode depend on the nature of this compound. Therefore, the main objective of the research was to develop a cheap, accurate electrode containing macrocyclic compounds for the determination of chlorhexidine in pharmaceuticals. Macrocyclic compounds with interesting structures and unique physical and chemical properties include an increasing number of natural compounds, such as valinomycin and cyclodextrins, and synthetic compounds, for instance, crown ethers, with the ability to selectively bind ions. Moreover, it can be noted that there are relatively few electrodes with crown ethers that have been developed for the purpose of determining medicinal substances [33,34] compared with electrodes sensitive to simple ions [35,36,37,38]. Cyclodextrins are a more popular group that forms complexes with various organic compounds used in potentiometric response mechanisms. In this numerous, not fully researched group of compounds, modified cyclodextrins deserve special attention. In order to improve their properties, e.g., complex-forming ones, cyclodextrins are subjected to various modifications of the hydroxyl groups. The alkylation of hydroxyl groups in positions 2, 3, and 6 results in a more lipophilic character for the cyclodextrins, which, in turn, allows for their application as potential ionophores in the membranes of ion-selective electrodes.

It can be assumed from the literature data that CHXD will form labile complexes with cyclodextrins [39]. In this new concept, solid-contact PVC membrane electrodes supporting two different macrocyclic compounds, dibenzo-18-crown-6 and heptakis (2,3,6-tri-O-benzoyl)-*β*-cyclodextrin and an ion exchanger from the borate salt group, were tested. 

Since cyclodextrins and crown ethers are neutral ionophores, a lipophilic salt, i.e., a borate anion with an opposite sign to the compound being determined, is required. The addition of a lipophilic salt with ion-exchanger properties provides permselectivity, stabilizes the charged complex in the membrane, prevents counter-ion coextraction from the sample, and maintains the constant concentration of the labeled ion in the membrane.

The influence of two ionophores and plasticizers, DOS and o-NPOE, on the characteristics of the electrodes and on the selected analytical parameters was studied. To assess the importance of the selected ionophores, other membranes with only the ion exchanger tetrakis [3,5-bis(trifluoromethyl)phenyl]borate were considered with respect to their potentiometric characteristics. The composition of the six constructed electrodes is presented in Table 1.

**Figure 3:**
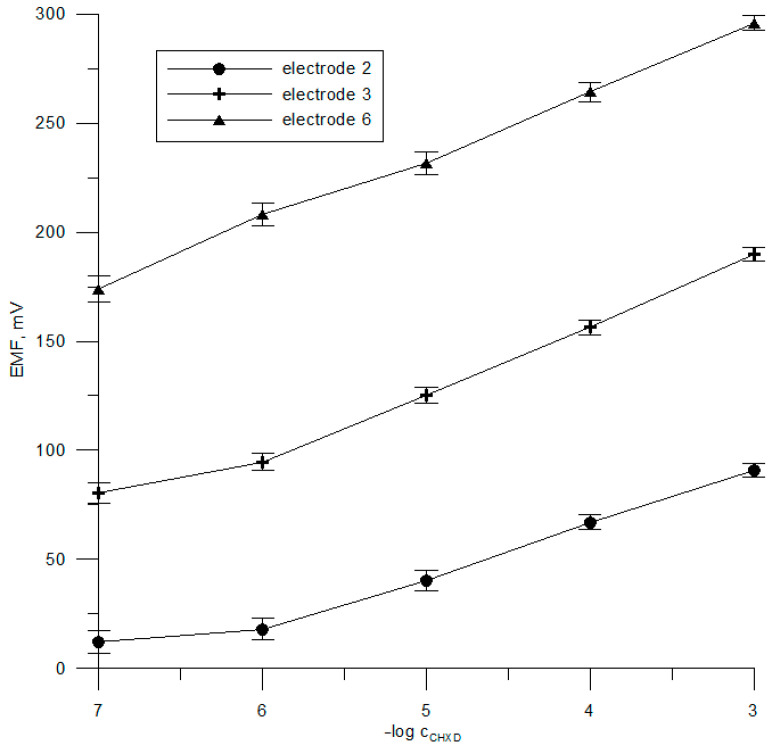
Chlorhexidine electrode calibration curve in chlorhexidine digluconate solutions.

The electrode response results are provided in Table 2 and Figure 3. It can be seen that the results for the three selected sensors with DBC-6, HSB*β*CD, and NaFBP, respectively, nos. 2, 3, and 6, demonstrate a near Nernstian response of about 26–30 mV decade^−1^ within 1 × 10^−6^–1 × 10^−3^ mol L^−1^ and a lower limit of detection of 2.5 × 10^−7^–5 × 10^−7^ mol L^−1^. The calibration graphs of electrodes 2, 3, and 6 have a good correlation coefficient of about 0.995–0.998. Lower sensitivities in the shorter linear range (1 × 10^−5^–1 × 10^−3^ mol L^−1^) were noticed for sensors 1, 4, and 5 with the same ionophores but different plasticizers. Thus, the lipophilicity, polarity, and chemical structure of the plasticizers exert a significant influence on the calibration graph parameters. The electrodes with the membrane plasticized with the o-NPOE with the highest polarity (dielectric constant ε = 14); with DBC-6 and NaFBP, they were more sensitive, while the potentiometric sensors prepared with HSB*β*CD plasticized with more lipophilic DOS, thus showing better performance. The effect of the plasticizer on the extraction characteristics of the polymer membrane is very clear.

### 3.2. Selectivity

Selectivity is one of the most important parameters of ion-selective electrodes. This value is determined with the complex formation constants of the cation and ionophore. In the case of electrodes containing an ion exchanger in the membrane, selectivity will depend on the partition coefficient of the cation in the water/organic membrane system. In some instances, obtaining a highly selective membrane can be conditioned by the complex formation constants of ionophores in suitable organic solvents. In the presence of macrocyclic compounds such as ionophores, the key factor contributing to molecular recognition in solution is the placement of the molecule in a hydrophobic cavity via spatial adjustment. However, the mechanism of molecular recognition in a membrane system introduces certain difficulties due to competitive reactions between membrane components. The overall selectivity of the electrode is, therefore, determined by the lipophilicity of the membrane, i.e., the effect of its other components, i.e., the plasticizer, the lipophilic salt, and the array [40]. 

In this study, the potentiometric selectivity coefficients of the chlorhexidine-selective electrodes were determined with respect to some inorganic cations, as well as additives and pharmaceutical excipients. The determinations were performed with the Separate Solution Method (SSM) [32]. Calibration curves in the concentration range of the main and interfering ion solutions (10^−5^–10^−3^ mol L^−1^) were recorded for this purpose. Then, the potentiometric selectivity coefficients were calculated with the interfering and main ions at 10^−3^ mol L^−1^ according to the following equation:

$$logK_{CHXD,N}^{pot}=\left(\frac{E_{2}-E_{1}}{S}\right)-\left(\frac{z_{CHXD}}{z_{N}}-1\right)logc_{CHXD}$$

where N is the interfering ion, E_2_ is the potential of the electrode in the interfering ion solution, E_1_ is the potential of the electrode in the main ion solution, S is the main ion slope of characteristics, z_CHXD_is the charge of the main ion (z_CHXD_ = 1), z_N_ is the charge of interfering ions, and c_CHXD_ = 1 × 10^−3^ mol L^−1^. 

From the data presented in Table 3, it can be seen that for certain ionic molecules, the K values are different. Very valuable and small values for the selectivity coefficients toward K^+^, Mg^2+^, sodium citrate, mannitol, glucose, and SDS were achieved for electrode no. 3. It is worth noting that for Na^+^, Ca^2+,^ glycerine, glycol, sucralose, and xylitol, the best results were obtained with electrode no. 6. It is impossible to clearly determine which sensor has the best selectivity. However, the obtained K values are favorable in all cases, and the tested ions and molecules should not influence the determination result in real samples.

The experimental data show that the optimal sensor electrode for chlorhexidine determination cannot be identified yet. All three selected electrodes provided a very similar potentiometric response. Therefore, further research is needed to determine other useful parameters. 

### 3.3. Reversibility, Response Time, and Electrode Drift

**Figure 4:**
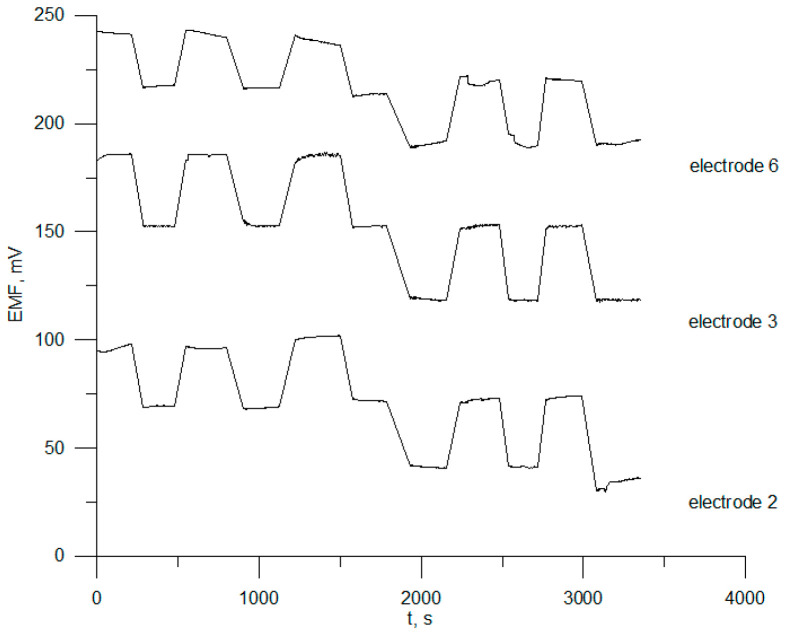
Reversibility of electrodes 2, 3, and 6.

To obtain reliable and accurate results for substance determinations using ion-selective electrodes and time-dependent parameters, including response times, potential reversibility and potential stability are often determined. In order to evaluate reversibility and potential, the selected electrodes, nos. 2, 3, and 6, were repeatedly immersed in chlorhexidine digluconate solutions at three different concentrations (1 × 10^−3^, 1 × 10^−4^, and 1 × 10^−5^ mol L^−1^). Figure 4 shows the results for one-month-old electrodes.

This figure shows that the average values of the potential of electrode no. 3 were 182 ± 0.7 mV, 153 ± 3.3 mV, and 117 ± 1.3 mV in CHXD solutions 1 × 10^−3^, 1 × 10^−4^, and 1 × 10^−5^ mol L^−1^, respectively. The relative standard deviation of the potential measurements was between 1.0% and 1.8%. For the other two sensors, it can be seen that the potential was not reversible. 

**Figure 5:**
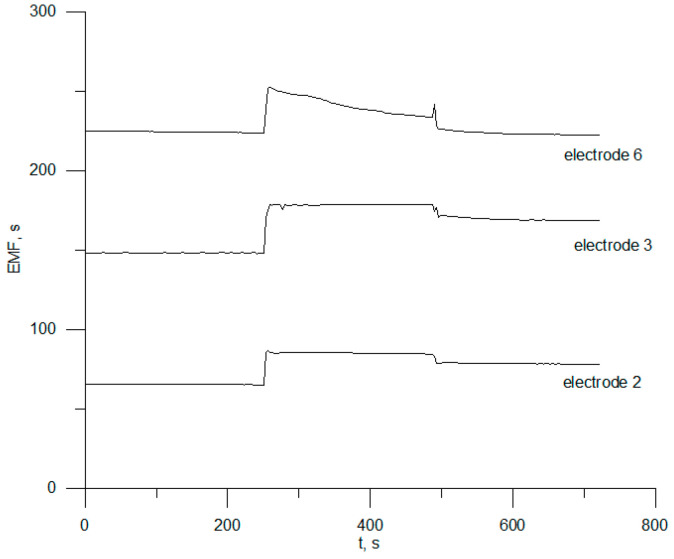
Response time of CHXD electrodes.

A method of injecting a concentrated standard solution (C_s_ = 10^−2^ mol L^−1^ vs. = 4 mL) into the intensively stirred chlorhexidine sample solution (C_CHXD_ = 10^−4^ mol L^−1^, V_CHXD_ = 50 mL) was used to determine the response time of the chlorhexidine electrodes (Figure 5).

When the potential was stable, the sample was diluted with deionized water in a ratio of one to one. The results shown in the figure provide information about the response time when the sample was concentrated, which was ~5–10 s and it was ~10 s when it was diluted.

To determine the short-term stability of chlorhexidine electrodes 2, 3, and 6, the potential was measured when the electrodes were immersed in 1 × 10^−4^ mol L^−1^ CHXD solution for 1 h. The potential drift determined from ΔE/Δt was 38 µV/min for electrode 2, 48 µV/min for electrode 3, and 0.4 mV/min for electrode 6.

**Figure 6:**
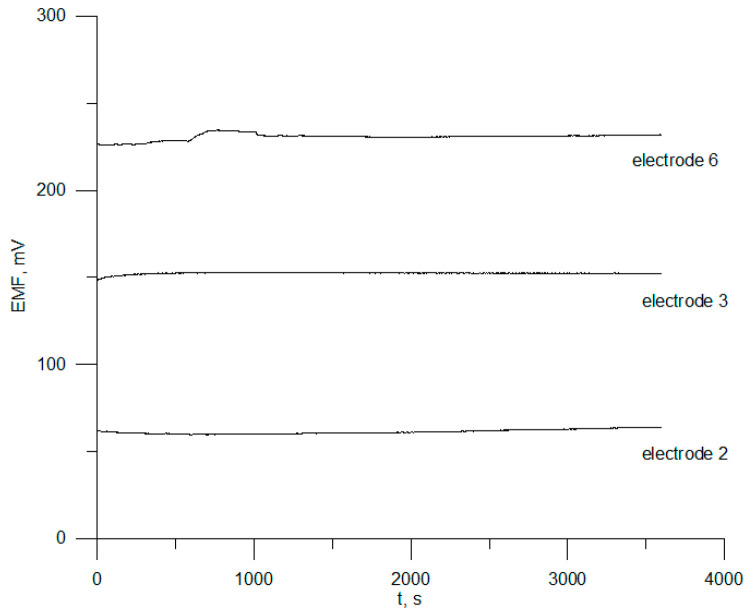
Electrode drift.

The electrodes were approximately 2 months old. The results obtained for the selected electrodes are shown in Figure 6. It can be seen that the most stable potential was achieved for sensors 2 and 3.

### 3.4. Working pH Range 

Measurements were made to determine the range in which the electrode potential is independent of the sample pH, the so-called working pH range. For the examined electrode, no. 3, it was determined according to a method described in earlier papers [41,42] using a solution of 10^−4^ mol L^−1^ CHXD and solutions of HCl and NaOH as additives. 

**Figure 7:**
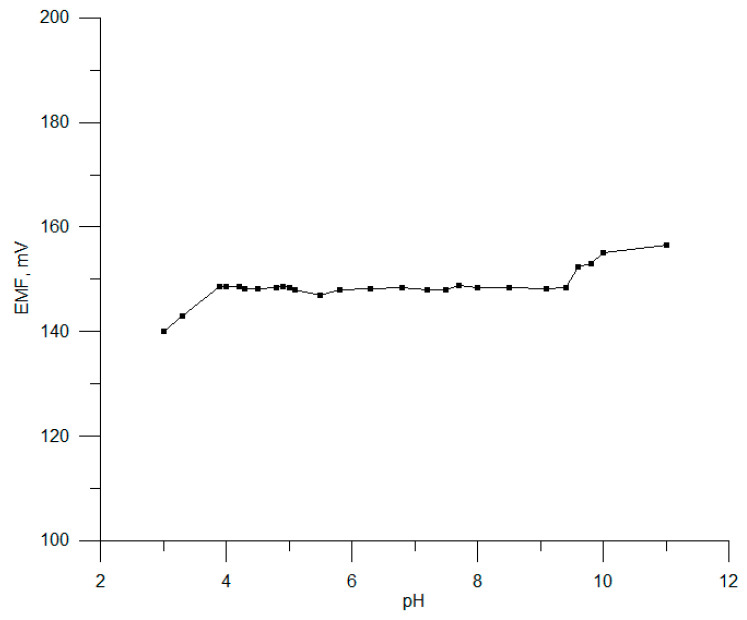
The effect of pH on CHXD electrode no. 3.

The potential dependence of the tested electrodes was measured in a pH range of 3–11, and it is shown in Figure 7. A stable potential response was found for pH 4.0 to 9.5. In an acidic medium (pH < 4), the interference effect from H^+^ ions is visible, while the increase that takes place at pH values higher than 9.5 is most likely the result of the formation of a free chlorhexidine base in the test solution.

### 3.5. Lifetime

**Figure 8:**
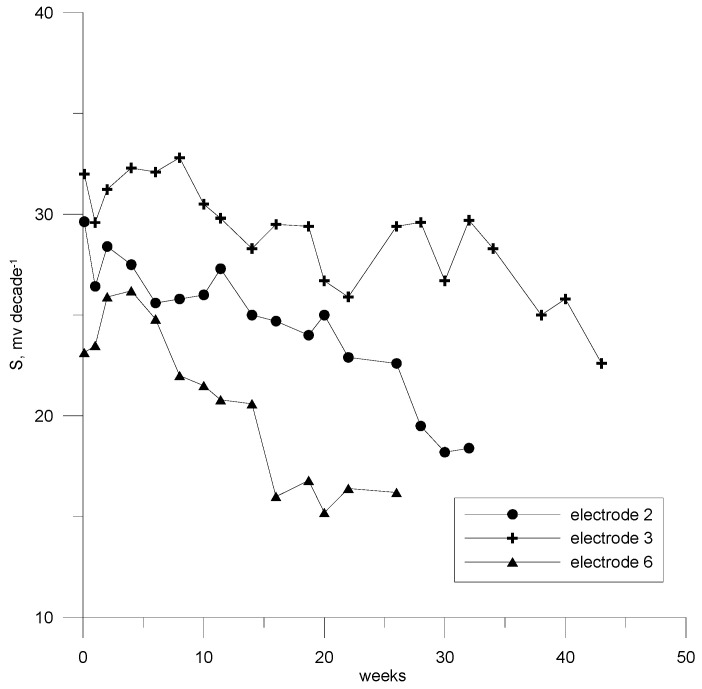
Time dependence of the characteristic slope of electrodes 2, 3, and 6.

The electrodes’ lifespans were examined by measuring the slope of their characteristics while they were kept at room temperature in air. The measurements were systematically taken, typically in 14-day intervals, using freshly prepared chlorhexidine digluconate solutions. The lifespans were determined up to the point where a deviation of ±16% from the Nernstian slope was detected [43]. The investigated electrode with HSB*β*CD achieved 10 months, the longest lifespan. The other electrodes tested in this work achieved the following lifespans: electrode no. 2—5 months; electrode no. 6—3 months. The results are presented in Figure 8. 

### 3.6. Analytical Application

The selected electrode (no. 3) with the best parameters was used to determine chlorhexidine digluconate in a pure sample and in pharmaceuticals. The analysis was conducted using the calibration curve, the standard addition method, and Gran’s method. As shown by the experimental data, typical results for the determination using ion-selective electrodes were achieved. The obtained values from three replicate measurements for the determination of chlorhexidine in clear solutions were 101% (RSD, 1.9–2.5%); in the pharmaceutical preparation Corsodyl, 99.5–95% (RSD 0.35–7.60%); and in Eludril Extra, 104.1–101.8 % (RSD 1.1–4.5%). The results are characterized by exactness and precision and make the electrode suitable for pharmaceutical and research laboratories. See Table 4.

## 4. Conclusions

A comparative study of Ag/AgCl solid contact electrodes based on macrocyclic compounds and lipophilic salt as active agents was presented. It was shown that all the electrodes containing the tested active compounds in the membrane, specifically, heptakis (2,3,6-tri-O-benzoyl)-*β*-cyclodextrin, dibenzo-18-crown-6, and sodium tetrakis [3,5-bis(trifluoromethyl)phenyl]borate, show a similar potentiometric response. Significant differences in the parameters, such as selectivity, potential reversibility, and stability, over time are clear. On this basis, it can be concluded that the best results were obtained for electrode no. 3. Particularly, the incorporation of lipophilic heptakis (2,3,6-tri-O-benzoyl)-*β*-cyclodextrin enabled a good sensor to be obtained, with a fast response time, the best potential stability, and a long lifespan (10 months). After this time, the sensor only requires a simple regeneration of the second membrane phase. It is then ready for use as if it were a new electrode. It is very easy to use the proposed electrode. This sensor is designed to be stored in the air and does not need to be stored in a vertical position. It is characterized by mechanical resistance and is a self-acting sensor. This electrode has a relatively long lifespan because of the design of the sensor. The absence of an internal reference solution limits the leaching of its membrane elements. Moreover, the electrode can be prepared quickly and easily from commonly available and cheap materials, unlike graphene or glassy carbon. It is worth mentioning that drug-sensitive electrodes are not commercially available and can only be obtained in the laboratory. The approximate price of only the materials used to construct the sensor presented in this paper is approximately 10 times less than the price of any other commercial ion-selective electrode.

## Figures and Tables

**Table 1:** Composition of the membranes of the chlorhexidine electrodes.

Qualitative and Quantitative Composition of the Outer Layers (mg)							
Number of Electrodes	PVC	DOS	o-NPOE	DBC	KTpClBP	HSBβCD	NaFBP
1	60	130	-	6	4	-	-
2	60	-	130	6	4	-	-
3	60	130	-	-	4	6	-
4	60	-	130	-	4	6	-
5	60	130	-	-	-	-	10
6	60	-	130	-	-	-	10

**Table 2:** The determined basic analytical parameters.

Electrode No. Membrane Composition	S ± s, mV Decade^−1^	E^0^, mV	R^2^	*n*	Linear Range, mol L^−1^	LD, mol L^−1^
1 DBC+KTpClBP+DOS	22.5 ± 5.8	279.6 ± 74.6	0.9959 ± 0.0048	5	1 × 10^−5^–1 × 10^−3^	3 × 10^−6^
2 DBC+KTpClBP+NPOE	26.7 ± 3.7	96.77 ± 77.0	0.9948 ± 0.0026	5	1 × 10^−6^–1 × 10^−3^	5 × 10^−7^
3 βCD+KTpClBP+DOS	30.4 ± 2.9	214.7 ± 40.6	0.9970 ± 0.0026	6	1 × 10^−6^–1 × 10^−3^	4 × 10^−7^
4 βCD+KTpClBP+NPOE	11.0 ± 3.2	477.6 ± 58.9	0.9972 ± 0.0027	5	1 × 10^−5^–1 × 10^−3^	2 × 10^−6^
5 NaFBP+DOS	16.0 ± 4.0	233.3 ± 85.5	0.9948 ± 0.0028	6	1 × 10^−5^–1 × 10^−3^	8 × 10^−6^
6 NaFBP+NPOE	26.8 ± 3.4	266.5 ± 79.0	0.9969 ± 0.0025	4	1 × 10^−6^–1 × 10^−3^	2.5 × 10^−7^

**Table 3:** Selectivity coefficients of the presented electrodes in comparison with other chlorhexidine electrodes.

$K_{CHXD/Interf}^{pot}$					
	Electrode 2	Electrode 3	Electrode 6	[24]	[23]
KCl	8.36	1.19 × 10^−1^	7.12 × 10^−1^	−	−
NaCl	2.33	1.08	7.60 × 10^−1^	2.57	−
CaCl_2_	1.46 × 10^−5^	4.03 × 10^−5^	2.28 × 10^−7^	3.02 × 10^−3^	6.81 × 10^−5^
MgCl_2_	4.84 × 10^−5^	3.98 × 10^−5^	1.28 × 10^−3^	3.02 × 10^−3^	4.28 × 10^−4^
Citrate	7.97 × 10^−6^	3.4 × 10^−8^	8.23 × 10^−3^	−	−
Mannitol	2.52 × 10^−5^	1.04 × 10^−7^	1.14 × 10^−1^	−	−
Glucose	9.39 × 10^−5^	1.05 × 10^−7^	4.56 × 10^−2^	−	−
SDS	6.42 × 10^−3^	2.23 × 10^−6^	5.69 × 10^−1^	−	1.36 × 10^−6^
Glycerine	1.79 × 10^−4^	1.48 × 10^−4^	1.21 × 10^−5^	−	−
Glycol	1.51 × 10^−4^	2.64 × 10^−4^	1.59 × 10^−5^	−	−
Sucralose	1.01 × 10^−4^	4.37 × 10^−4^	4.09 × 10^−5^	−	−
Xylitol	1.93 × 10^−3^	1.47 × 10^−3^	3.39 × 10^−4^	−	−

**Table 4:** Results obtained from the analysis of CHXD samples using the proposed ion-selective electrode, no. 3 (*n* = 3).

Sample	Method	Taken mol L^−1^	Found mol L^−1^	Relative Error, %	RSD, %	Confidence Range, mol L^−1^
Pure (Alfa Aesar)	Calibration Curve	1.00 × 10^−4^	1.01 × 10^−4^	0.76	1.89	1.01 × 10^−4^ ± 3 × 10^−6^
	Standard Addition	1.00 × 10^−4^	1.01 × 10^−4^	1.04	2.49	1.01 × 10^−4^ ± 6 × 10^−6^
Corsodyl (GlaxoSmith)	Calibration Curve	2.22 × 10^−4^	2.19 × 10^−4^	1.15	0.35	2.19 × 10^−4^ ± 2 × 10^−6^
	Standard Addition	2.22 × 10^−4^	2.09 × 10^−4^	5.81	7.60	2.09 × 10^−4^ ± 3.9 × 10^−5^
Eludril Extra (Pierre Fabre)	Calibration Curve	2.22 × 10^−4^	2.29 × 10^−4^	3.19	4.87	2.29 × 10^−4^ ± 2.7 × 10^−5^
	Gran’s Method	2.22 × 10^−4^	2.24 × 10^−4^	1.20	1.12	2.24 × 10^−4^ ± 6 × 10^−6^

## Data Availability

Data are contained within the article.
